# Protein-to-carbohydrate ratio is informative of diet quality and associates with all-cause mortality: Findings from the National Health and Nutrition Examination Survey (2007–2014)

**DOI:** 10.3389/fpubh.2022.1043035

**Published:** 2022-12-22

**Authors:** Therese M. C. Wabo, Yifeng Wang, Rose M. Nyamao, Wenjie Wang, Shankuan Zhu

**Affiliations:** ^1^Chronic Disease Research Institute, The Children's Hospital, National Clinical Research Center for Child Health, School of Public Health, School of Medicine, Zhejiang University, Hangzhou, China; ^2^Department of Nutrition and Food Hygiene, School of Public Health, Zhejiang University, Hangzhou, China; ^3^Department of Medical Microbiology and Parasitology, School of Medicine, Kenyatta University, Nairobi, Kenya; ^4^Department of Microbiology, School of Basic Medical Sciences, Harbin Medical University, Harbin, China

**Keywords:** protein-to-carbohydrate ratio, diet quality, Healthy Eating Index, Total Nutrients Index, all-cause mortality

## Abstract

**Background:**

Dietary protein and carbohydrate intake and health outcomes have received extensive attention in recent years. However, the nutritional context in which these associations occur is less studied.

**Objectives:**

We aimed to examine the dietary context associating protein-to-carbohydrate ratio and all-cause mortality in US adults.

**Methods:**

Data from 17,814 adults enrolled in the 2007–2014 NHANES was analyzed. Information on mortality was obtained from the US mortality registry updated in December 2015. Diet quality was assessed using the Healthy Eating Index (HEI) and Total Nutrients Index (TNI). ANCOVA was used to test the mean differences in HEI and TNI scores across %E P:C quintiles. Linear regression examined the association of HEI and TNI with %E P:C. Cox proportional hazards regression evaluated the association between %E P:C and all-cause mortality. A restricted cubic spline examined the non-linear relationship between %E P:C and death.

**Results:**

Low %E P:C was associated with lower HEI and TNI scores while higher %E P:C was associated with healthier HEI and TNI scores. HEI and TNI were positively associated with %E P:C (*β* = 0.22, 95% CI: 0.19–0.25, and *β* = 0.16, 95% CI: 0.14–0.18), respectively. Low %E P:C was associated with an increased risk of death from all-cause. The higher HRs (95% CIs) of all-cause mortality were 1.97(1.46–2.65), and 7.35 (2.57–21.03) in the second quintile for the age-sex-ethnicity model, and the fully adjusted model, respectively. There was a significant reverse *U*-shape relationship between %E P:C and all-cause mortality with *P*, non-linearity < 0.001.

**Conclusion:**

This study indicates that a low %E P:C that gives emphasis to unhealthy foods increases the risk of death. Hence, it would be useful to consider the complete diet associated with protein intake when making dietary recommendations for populations.

## Introduction

Diet, as a modifiable lifestyle factor, has shown benefits in the prevention and management of chronic disease and premature death (1, 2). Adequate protein intake is one of the vital nutritional factors essential for a healthy and well-functioning human body. High protein intake with restriction of energy from carbohydrates has received extensive attention in recent years. Evidence has revealed the health benefits of a high-protein diet for weight loss and management of cardiometabolic risks, including improving glycemic control in people with diabetes mellitus, reducing blood pressure, and maintaining metabolic parameters (3, 4). Conversely, long-term observational studies have shown that diets high in protein, are associated with an unfavorable risk of type 2 diabetes, hypertension, and metabolic syndrome (5, 6). A constant high-protein diet has been found to increase glucagon and insulin stimulation, probably decreasing insulin sensitivity (7–9). These associations between protein consumption and health outcomes remain inconsistent in nutrition research since the findings of observational and experimental studies are conflicting (3–11). However, consuming enough protein while reducing carbohydrate intake, especially caloric-dense carbohydrates, remains a constant recommendation in many countries like the US, China, and England, for human growth, development, and health (12–15). The gaps in the findings regarding protein and carbohydrate intake could be due to a biased focus on their absolute value, the protein and carbohydrate source against health outcomes ignoring the complete diet associated with protein and carbohydrate intake (6, 10, 16).

Consequently, this study aimed to examine the dietary context associating protein-to carbohydrate percentage energy ratio with all-cause mortality. To achieve our aim, we assessed the relationship between protein-to-carbohydrate percentage energy ratio (%E P:C) and overall diet quality assessed by Healthy Eating Index (HEI-2015) and the Total Nutrients Index (TNI); and examine the corresponding association with all-cause mortality among US adults using four cycles of the National Health and Nutrition Examination Survey (NHANES).

## Methods

### Study population

The NHANES collects health information from representative samples of the American population through interviews, medical examinations, and laboratory tests. The survey results are used to determine the prevalence rate and risk factors of major diseases, help to formulate public health policies, design health programs, and services, and expand national health knowledge.

Included in this study are American adults who participated in the NHANES from 2007 to 2014. The data consisted of 40,617 participants, including men and women of all ages. All participants completed a self-management questionnaire, including lifestyle, socio-demographic factors, health-related diseases, etc. After exclusion of participants aged <18 years (*n* = 15,885), with unreliable information on the first and second 24-h dietary recall (*n* = 4,411), missing information on macronutrients intake (*n* = 838), pregnant women (*n* = 317), kidney failure (*n* = 558), and total energy intake ≥4,200 or ≤ 500 kcal/d (*n* = 794), a total of 17,814 participants, including 8,366 men and 9,448 women, were included in this study.

### Exposure and outcome

The main exposure is the protein-to-carbohydrate percentage energy ratio (%E P:C). Dietary protein and carbohydrate intake were measured by a 2-days 24-h dietary recall method administered by trained interviewers at a mobile examination center (MEC). The NHANES Day 2 dietary recall was collected by telephone ~3–10 days after the MEC exam. The recall data included all foods and beverages consumed by subjects within a 24-h period. The US Department of Agriculture (USDA), Food and Nutrient Database for Diet Studies (FNDDS) assigned nutrient values to foods (17). Daily carbohydrate and protein intakes were transformed as percentage of total energy intake by assuming the energy value of 4 kcal/g for carbohydrates and 4 kcal/g for proteins using the percentage of each macronutrient from total calories. The %E P:C was obtained after dividing the percentage of energy from protein intake by the percentage energy from carbohydrates ($\%\;E\;P:C=\frac{Protein\;percentage\;energy}{carbohydrate\;percentage\;energy}$).

The outcome variable was mortality status extracted from the NHANES Public-use Linked Mortality Files. The vital status and cause of death was determined by the National Death Index by December 31, 2015. All-cause mortality included all specified and unknown causes (18). In total, 1,026 deaths were documented. The linked morality data is available at: ftp.cdc.gov-/pub/Health_Statistics/NCHS/datalinkage/linked_mortality/.

### Diet quality

The food intake quality was assessed using the Healthy Eating Index (HEI)-2015, which measures the alignment of the dietary intakes of a set of foods within the 2015–2020 Dietary Guidelines for Americans (DGA) (19). The HEI-2015 includes 13 graded components on a 100-point scale for maximum score. This scale assesses amounts per 1,000 kcal with higher scores on each subscale. The higher total score represents better diet quality related to the DGA. Food components were extracted from the Food Patterns Equivalents Database (FPED) of the What We Eat in America **(**WWEIA) and the Food and Nutrient Database for Dietary Studies (FNDDS).

The total micronutrients intake was assessed using the newly developed Total Nutrients Intake (TNI), an index designed to describe the usual intake from all sources of under-consumed micronutrients among the US population. The TNI assesses the US adults' total nutrient intakes relative to recommended nutrient standards for eight under-consumed micronutrients identified by the DGA, i.e., calcium, magnesium, potassium, choline, vitamins A, C, D, and E. TNI extends existing measures of diet quality by including nutrient intake from all sources. For each individual, the ratio of their total usual nutrient intake (diet and supplements) to the corresponding age-and-sex-specific Recommended Dietary Allowance (RDA), and Adequate Intake (AI), is scored with truncation at a maximum ratio of 1.0, and the ratio is multiplied by 100. The score of 8 components is then averaged to yield the TNI score. A high score represents a good adherence to the RDA or AI as described in the DGA. Details on the TNI score can be found elsewhere (20).

### Covariates

Dietary variables included total energy (kcal/d); fat intake transformed as %E of total energy intake by assuming a mean energy value of 9 kcal/g for fat intake. Sodium intake was obtained from the average total dietary and supplement intake for the 2 days of 24-h recalls.

Non-dietary variables included age, sex, ethnicity, family income, highest education level, body mass index (BMI), and SBP (Systolic Blood Pressure). SBP was used as a confounder for its strong association with diet and cardiovascular outcomes (21, 22). SBP was the average of three readings obtained under standard conditions during a single physical examination. Alcohol consumption, smoking status (yes/no), and physical activity level (PAL); Alcohol consumption was defined as heavy consumption if self-reported consumption is more than two drinks per day for men and more than one drink per day for women; and mild consumption if less (23). Physical activity was measured using questionnaires that collected the time spent in all activities in a typical week, including work, leisure, travel, and household chores. Physical activity level (PAL) was assessed using self-reported measures converted into metabolic equivalents (Mets). A summary measure expressed as total Mets was created by multiplying the time spent in each activity with the Mets of each activity. The time spent in each activity was converted into the Mets min/hours per week based on the compilation of physical activities (24). Mets was then divided into three levels “low PAL” < 600 Mets min/week, “moderate PAL” 600–1,200 Mets min/week, and “high PAL” ≥ 1,200 Mets min/week. Since diabetes and hypertension are nutrition-related diseases and are among the leading causes of death in the US, being under hypertension and/or diabetes medication were considered confounders.

### Statistical analysis

Statistical analyses accounted for 8 years of sample weights and all other complex domains in the NHANES analytic guidelines (25). The %E P:C ratio was transformed into z-scores to examine the effect of a 1 SD exposure change on outcomes. The %E P:C *z*-scores were divided into quintiles, with the first quintile (Q1) representing the lowest fifth with the lower protein and higher carbohydrate intake and the fifth quintile (Q5) representing the highest fifth with higher protein and lower carbohydrate intake.

Demographic characteristics, anthropometric measurements, and macronutrient intake were presented as mean (±SE) for continuous variables and percentage for categorical variables. Chi-square and ANOVA (analysis of variance) were used to compare the baseline characteristics by quintiles.

Analysis of covariance (ANCOVA) was performed to compare the mean differences in HEI and TNI components scores across %E P:C quintiles controlled by age, sex, education level, family income, BMI, and total energy intake. We carried out a multivariable linear regression to assess the association of HEI and TNI with the %E P:C ratio. The regression was adjusted for age, sex, education level, family income, BMI, and total energy intake.

Cox proportional hazards regression was conducted to investigate the association of %E P:C with all-cause mortality. Three regression models controlling for various confounding factors were constructed: the unadjusted model (model 1); the age, sex, and ethnicity adjusted model (model 2); and model 3 additionally adjusted for education level, family income, BMI, Mets, alcohol consumption, smoking status, diabetes medications, hypertension medications, sodium intake, SBP, total energy, and fat percentage energy. BMI and Mets were adjusted as continuous variables.

To remove the driven effect of carbohydrates in the %E P:C association, we further assessed the association between %E Protein (%E P) and all-cause mortality. In addition to the two models mentioned above, we built an isocaloric regression model with %E P as exposure, adjusting for fat while excluding carbohydrates. In the carbohydrate-restricted model, we examined a 1:1 kcal by including all energy-contributing nutrients, except for carbohydrates, in an isocaloric model (26). In such a model, the estimated relationship with outcomes could be interpreted as increasing protein intake at the expense of carbohydrates while keeping calories constant.

We performed a restricted cubic spline (RCS) with three knots at the 5th, 50th, and 95th percentiles, adjusted for variables in the third model to determine the non-linear relationship between %E P:C, %E P and all-cause mortality.

For the sensitivity analysis, adults younger than 30 years with a follow-up time <3 years or those who died within 3 years of follow-up were further excluded.

All statistical analyses were conducted using STATA version 13.0 and R-project 4.1.2 with a 2-sided *P* < 0.05 considered statistically significant.

## Results

In the current study, 17,814 participants were included (mean ± SE age: 46.15 ± 0.33 years; 52.8% female). Table 1 shows the baseline characteristics of the study population according to %E P:C quintiles. Participants in the lowest %E P:C quintile tended to be younger non-Hispanic white smokers who are highly active, with high educational attainment and the lowest BMI. They also tended to have the lowest energy intake of protein and fat, the second lower total energy intake, and the highest energy intake from carbohydrates. Participants with the highest %E P:C were likely to be older non-Hispanic whites, with the second highest BMI, higher educational attainment and family income, non-smokers, highly active, with the lowest energy intake from carbohydrates and the highest energy intake from protein and fat.

**Table 1:** Baseline characteristics of the study population by %E P:C *z*-score quintiles.

**Variables**	**Q1 (*n* = 3,563)**	**Q2 (*n* = 3,563)**	**Q3 (*n* = 3,563)**	**Q4 (*n* = 3,563)**	**Q5 (*n* = 3,562)**	***p*-value**
**Demographic variables**						
Age	44.67 ± 0.29	45.91 ± 0.30	45.45 ± 0.29	46.99 ± 0.28	47.48 ± 0.27	< 0.001
**Sex (%)**						< 0.001
Male	41.76	45.78	48.62	50.19	49.01	
Female	58.24	54.22	51.38	49.81	50.99	
**Ethnicity (%)**						< 0.001
Mexican American	7.72	10.78	10.04	8.93	6.46	
Non-Hispanic white	65.61	65.21	66.73	67.32	73.15	
Non-Hispanic black	13.65	12.27	10.95	11.21	8.95	
Other Hispanic	5.42	5.85	5.48	5.14	5.00	
Non-Hispanic Asian	2.45	2.46	3.00	2.68	2.80	
Other race	5.14	3.43	3.78	4.72	3.61	
**Family income**						< 0.001
< $20,000	23.6	20.43	20.04	18.27	12.95	
$20,000–$44,999	30.54	28.57	26.61	26.48	23.42	
$45,000–$64,999	14.65	14.8	14.14	14.15	14.98	
>$65,000	31.21	36.2	39.21	41.10	48.65	
**Smoking status (%)**						0.06
Yes	46.61	42.54	41.12	43.85	43.82	
No	53.39	57.46	58.88	56.15	56.18	
**Alcohol (%)**						0.98
Mild consumption	49.29	48.43	48.48	49.10	49.44	
Heavy consumption	50.71	51.57	51.52	50.90	50.56	
**Education (%)**						< 0.001
9–11 grade	16.14	13.54	13.46	12.38	8.71	
High school diploma/GED	27.37	26.34	23.26	23.68	19.76	
Some college/associate	32.24	31.83	33.49	31.14	33.08	
College graduate or above	24.25	28.29	29.79	32.81	38.45	
**Anthropometric variables**						
BMI	28.11 ± 0.11	28.51 ± 0.11	28.63 ± 0.11	29.26 ± 0.12	29.08 ± 0.11	< 0.001
**Physical activity levels (min/wk.)** ^**a**^						< 0.001
METs	2,689.96 ± 109.53	2,431.57 ± 61.75	2,447.99 ± 127.12	2,460.85 ± 145.64	2,483.20 ± 131.59	< 0.001
Low	355.9 (16.92)	352.6 (19.58)	350.3 (17.82)	353.1 (19.46)	368.2 (17.4)	
Moderate	861.8 (21.38)	833.39 (21.63)	859.68 (24.45)	870.10 (24.04)	851.9 (23.75)	
Highly	3,963.9 (61.69)	3,711.8 (58.79)	3,768.2 (57.73)	3,865.7 (56.46)	3,766.9 (58.85)	
SBP	120.55 ± 0.28	120.74 ± 0.28	120.59 ± 0.28	122.11 ± 0.28	121.46 ± 0.28	< 0.001
DBP	69.76 ± 0.20	69.81 ± 0.19	70.18 ± 0.20	70.89 ± 0.20	70.41 ± 0.19	< 0.001
**Macronutrients**						
%E Protein	11.46 ± 0.03	14.30 ± 0.02	16.27 ± 0.03	18.48 ± 0.04	23.43 ± 0.08	< 0.001
%E Carbohydrate	59.96 ± 0.11	53.72 ± 0.09	50.17 ± 0.09	46.23 ± 0.09	37.88 ± 0.12	< 0.001
%E Fat	28.57 ± 0.11	31.96 ± 0.11	33.55 ± 0.12	35.27 ± 0.12	38.68 ± 0.14	< 0.001
Energy (kcal)	1,891.25 ± 12.32	1,972.17 ± 12.19	1,899.95 ± 11.91	1,893.04 ± 12.53	1,639.02 ± 11.62	< 0.01
P:C *z*-score	−0.80 ± 0.003	−0.42 ± 0.001	−0.11 ± 0.001	0.28 ± 0.002	1.62 ± 0.03	< 0.001
%E P:C	0.19 ± 0.0005	0.26 ± 0.0002	0.32 ± 0.0002	0.40 ± 0.0004	0.65 ± 0.006	< 0.001
Protein/kg/day	0.75 ± 0.005	0.94 ± 0.006	1.01 ± 0.006	1.10 ± 0.007	1.22 ± 0.007	< 0.001

Continuous variables are presented as weighted mean ±SE, weighted percentages for categorical variables.

a Mean (% of total).

### Diet quality and Protein-to-carbohydrate ratio

Differences in HEI components across %E P:C quintiles are presented in Table 2. There was a significant difference in HEI component scores across all quintiles after adjusting for age, sex, education level, family income, BMI, and total energy except for whole fruits, whole grains, fatty acids, and saturated fat (*p*-value >0.05). A lower %E P:C is associated with an overall unhealthy HEI score, and a higher %E P:C is associated with a healthier HEI score (*p*-value < 0.05). A lower %E P:C was significantly associated with a poor HEI score for total vegetables, greens & beans, dairy, protein foods, seafood and plant proteins, refined grains, and added sugars intake. A higher %E P:C was significantly associated with a wholesome HEI for the same food components (all *p*-value < 0.05).

**Table 2:** Differences in Healthy Eating Index 2015 scores across %E P:C *z*-score quintiles.

	**Q1 (*n* = 3,563)**	**Q2 (*n* = 3,563)**	**Q3 (*n* = 3,563)**	**Q4 (*n* = 3,563)**	**Q5 (*n* = 3,562)**	***p*-value**
**HEI-score components**						
Total fruits (0–5)	2.86 (0.03)	2.87 (0.03)	2.61 (0.03)	2.51 (0.03)	2.33 (0.03)	< 0.001
Whole fruits (0–5)	1.04 (0.03)	1.09 (0.03)	1.00 (0.03)	1.03 (0.03)	1.03 (0.03)	0.39
Total vegetables (0–5)	3.56 (0.03)	3.88 (0.03)	3.94 (0.03)	4.06 (0.03)	4.10 (0.03)	< 0.001
Greens and beans (0–5)	1.29 (0.03)	1.44 (0.03)	1.48 (0.03)	1.60 (0.03)	1.73 (0.03)	< 0.001
Whole grains (0–10)	3.14 (0.06)	3.19 (0.06)	3.22 (0.05)	3.07 (0.05)	3.04 (0.05)	0.15
Dairy (0–10)	4.95 (0.05)	5.51 (0.05)	5.91 (0.05)	5.92 (0.05)	6.06 (0.05)	< 0.001
Protein foods (0–5)	2.23 (0.01)	2.86 (0.01)	2.36 (0.01)	3.88 (0.01)	4.49 (0.01)	< 0.001
Seafood and plant proteins (0–5)	0.92 (0.02)	1.08 (0.02)	1.22 (0.02)	1.40 (0.02)	1.71 (0.02)	< 0.001
Fatty acids (0–10)	8.36 (0.05)	8.42 (0.05)	8.34 (0.05)	8.51 (0.05)	8.48 (0.05)	0.19
Refined grains (0–10)^a^	5.16 (0.06)	5.02 (0.06)	5.07 (0.06)	5.52 (0.06)	6.11 (0.06)	< 0.001
Sodium (0–10)^a^	5.31 (0.05)	4.04 (0.05)	3.26 (0.05)	2.84 (0.05)	2.47 (0.05)	< 0.001
Added sugars (0–10)^a^	3.53 (0.05)	5.42 (0.05)	6.32 (0.05)	7.14 (0.05)	7.62 (0.05)	< 0.001
Saturated fat (0–10)^a^	10 (1.25e−14)	10 (1.25e−14)	10 (1.25e−14)	10 (1.25e−14)	10 (1.25e−14)	0.5
Total HEI-score (0–100)	52.4 (0.19)	54.8 (0.19)	55.7 (0.18)	57.5 (0.18)	59.2 (0.18)	< 0.001

Mean (SE) intakes adjusted for age, sex, education level, family income, BMI, and total energy.

a Higher scores represent lower intake.

Table 3 shows the difference in nutrient intake assessed *via* TNI across %E P:C quintiles. After adjusting for age, sex, education level, family income, BMI and total energy, there was a significant difference in all TNI components across all quintiles (all *p*-value <0.05) for %E P:C. Adults in the lowest quintile had the lowest score and lowest intake of all eight nutrients of TNI (Calcium, Magnesium, Potassium, Choline, Vitamin A, Vitamin C, Vitamin D, and Vitamin E) for a TNI-score of 56.4 (0.25). In contrast, those in the higher quintile had a higher intake and better TNI-score 68.0 (0.24) for the same nutrients.

**Table 3:** Differences in Total Nutrient Index scores across %E P:C *z*-score quintiles.

	**Q1 (*n* = 3,563)**	**Q2 (*n* = 3,563)**	**Q3 (*n* = 3,563)**	**Q4 (*n* = 3,563)**	**Q5 (*n* = 3,562)**	***p*-value**
**TNI components**						
Calcium	66.6 (0.38)	71.5 (0.38)	73.3 (0.37)	73.8 (0.37)	74.3 (0.37)	< 0.001
Magnesium	78.2 (0.42)	82.4 (0.42)	82.4 (0.42)	84.5 (0.41)	86.5 (0.41)	< 0.001
Potassium	44.6 (0.28)	47.5 (0.28)	49.3 (0.28)	51.7 (0.28)	54.3 (0.28)	< 0.001
Choline	48.3 (0.32)	57.8 (0.32)	63.1 (0.31)	68.4 (0.31)	76.9 (0.31)	< 0.001
Vitamin A	60.0 (0.48)	64.6 (0.48)	66.4 (0.48)	67.3 (0.48)	70.1 (0.48)	< 0.001
Vitamin C	72.8 (0.57)	75.4 (0.57)	73.1 (0.56)	73.2 (0.56)	73.7 (0.56)	0.01
Vitamin D	34.8 (0.55)	39.0 (0.55)	41.7 (0.55)	43.4 (0.54)	52.3 (0.54)	< 0.001
Vitamin E	46.1 (0.38)	49.5 (0.38)	50.5 (0.38)	52.6 (0.37)	55.8 (0.38)	< 0.001
TNI total score	56.4 (0.25)	61.0 (0.25)	62.5 (0.24)	64.4 (0.24)	68.0 (0.24)	< 0.001

Mean (SE) intakes adjusted for age, sex, education level, family income, BMI, and total energy.

### Linear regression

Table 4 shows the association between TNI, HEI, and %E P:C. After adjusting for confounding variables, there was a significant positive association between TNI, HEI, and %E P:C. For a unit increase of %E P:C *z*-score, HEI increases by (ß = 0.22; 95% CI: 0.19–0.25) and TNI by (ß =0.16; 95% CI: 0.14–0.18) indicating its positive correlation with diet quality.

**Table 4:** Association between HEI-2015, TNI, and %E P:C ratio.

	**Standardized ß coefficient**	**ß coefficient 95% CI**	***p*-value**
Per one unit increase			
**HEI-2015**			
Crude	0.21	0.19–0.24	< 0.001
Adjusted^a^	0.22	0.19–0.25	< 0.001
**TNI**			
Crude	0.08	0.06–0.10	< 0.001
Adjusted^a^	0.16	0.14–0.18	< 0.001

Linear regression models.

a Adjusted for age, sex, education level, family income, BMI, and total energy.

### Cox proportional hazards models

We fitted three cox regression models to assess the association of %E P:C and %E P with all-cause mortality (Table 5). After multivariable adjustment, low %E P: C was associated with an increased risk of death from all-cause. Compared to the highest quintile (Q5), the higher HRs (95% CIs) of all-cause mortality were 1.97 (1.46–2.65) and 7.35 (2.57–21.03) in the second quintile (Q2) for the second and third models, respectively.

**Table 5:** Hazard ratio (95% confidence interval) of all-cause mortality for %E P:C *z*-score quintiles.

	**Model 1**		**Model 2**		**Model 3**		
	**HR**	**95% CI**	**HR**	**95% CI**	**HR**	**95% CI**
Q1	1.80	1.40–2.30	1.90	1.50– 2.41	1.84	0.57–5.86
Q2	2.02	1.48–2.75	1.97	1.46– 2.65	7.35	2.57– 21.03
Q3	1.49	1.14–1.96	1.60	1.19– 2.14	4.38	1.66–11.57
Q4	1.62	1.23–2.14	1.58	1.20– 2.10	1.99	0.69–5.74
Q5	1	Ref	1	Ref	1	Ref

Model 1: Unadjusted.

Model 2: Age, sex, and ethnicity.

Model 3: Model 2 + Education level, family income, BMI, METs, alcohol consumption, smoking status, diabetes medications (yes/no), hypertension medication, SBP, sodium intake, fat percentage energy, and total energy.

%E P assessed as exposure with all-cause mortality is shown in Table 6. Likewise, low protein intake was associated with an increased risk of all-cause mortality. Compared to the highest quintile, the higher HRs (95% CIs) of all-cause mortality were 1.72 (1.24–2.38) in the second model and 2.45 (1.12–5.36) in the isocaloric model.

**Table 6:** Hazard ratio (95% confidence interval) of all-cause mortality for %E P *z*-score quintiles + isocaloric regression model.

	**Model 1**		**Model 2**		**Isocaloric Model**	
	**HR**	**95% CI**	**HR**	**95% CI**	**HR**	**95% CI**
Q1	1.36	1.01–1.85	1.52	1.14–2.01	1.44	0.62–3.32
Q2	1.51	1.11–2.07	1.72	1.24–2.38	2.45	1.12–5.36
Q3	1.45	1.09–1.93	1.44	1.06–1.95	2.25	1.10–4.59
Q4	0.89	0.65–1.22	0.98	0.70–1.37	1.52	0.74–3.12
Q5	1	Ref	1	Ref	1	Ref

Model 1: Unadjusted.

Model 2: Age, sex, and ethnicity.

Isocaloric model: Model 2 + Education level, family income, BMI, METs, alcohol consumption, smoking status, diabetes medications, hypertension medication, SBP, sodium intake, fat percentage energy, and total energy.

Supplementary Tables 1, 2 show results from the sensitivity analysis. After exclusion of adults aged below 30 years, with a follow-up time <3 years or who died within 3 years of follow-up, the association of %E P:C and %E P with all-cause mortality yielded similar results in all models.

**Figure 1:**
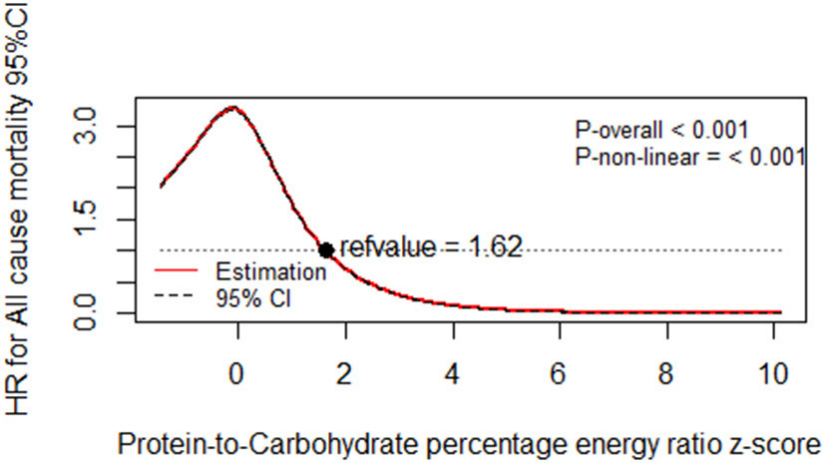
Dose-response relationship of protein-to-carbohydrate percentage energy ratio *z*-score with all-cause mortality. Multivariable-adjusted restricted cubic spline analysis for the continuous association of %E P:C and all-cause mortality. Covariables are listed in the Table 5 footnotes (model 3). The *y-*axis shows the HRs for all-cause mortality for any %E P:C intake *z*-score, compared with the reference value set at an intake of 1.62. The black dot line indicates the 95% CIs.

**Figure 2:**
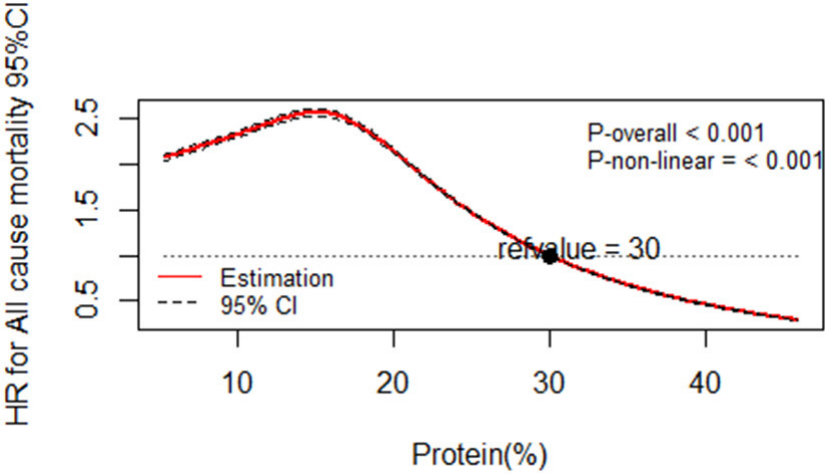
Dose-response relationship of protein percentage energy with all-cause mortality. Multivariable-adjusted restricted cubic spline analysis for the continuous association of %E P and all-cause mortality. Covariables are listed in the Table 6 footnotes (isocaloric model). The *y*-axis shows the HRs for all-cause mortality for any %E P intake compared with the reference value set at 30% energy intake from protein. The black dot line indicates the 95% CIs.

Illustrated according to the restricted cubic spline models, Figures 1, 2 represent a cubic spline of %E P:C z-score, %E P with all-cause mortality. After adjusting for all covariates in the third model, there was a significant reversed U-shaped relationship between the %E P: C, %E P, and all-cause mortality, enlightening the high risk of death in the low %E P: C ratio (*P* for non-linearity < 0.001) and low %E P (*P* for non-linearity < 0.001).

## Discussion

In the current study, the nutritional context associating %E P:C with diet quality and the corresponding association with all-cause mortality was examined. HEI and TNI were positively associated with %E P:C. For a unit increase of %E P: C, the HEI and TNI increased. Adults with low %E P:C, had poorer HEI and TNI scores, and high %E P:C with healthier HEI and TNI scores. Unhealthy low %E P:C was associated with an increased risk of death from all-cause with a significant reverse *U*-shape relationship.

Previously, a moderate relationship between HEI-2015 and TNI has been reported and the two have been identified as useful tools reflecting population-level adherence to nutrient standards of total food intakes and under-consumed micronutrients in the US (27). The %E P:C assessed with diet quality has received less attention. This is the first study to assess dietary patterns associated with the %E P:C ratio using HEI-2015 and TNI. Concerning the link between proteins and carbohydrates with health outcomes, it is often assumed that its positive or negative effects on health depend on their source, the total intake, or their percentage of energy (28–30). However, these associations may appear in the context of a healthy or unhealthy diet.

In our study, adults with Low %E P:C were more likely to have a lower intake of vegetables, greens and beans, dairy, protein foods, seafood and plant proteins, a higher intake of refined grains, added sugars, with lower calcium, magnesium potassium choline vitamin A vitamin C vitamin D and Vitamin E intake. In contrast, adults with high %E P:C tended to have a high intake of vegetables, greens and beans, dairy, protein foods, seafood and plant proteins, lower intake of refined grains, added sugars, high calcium, magnesium, potassium, choline, vitamin A, vitamin C vitamin D, and Vitamin E intake.

Low protein intake is usually high in carbohydrates, especially caloric-dense carbohydrates (31); this is consistent with our findings wherein adults with low %E P: C showed a diet high in refined grains and sugar. According to the Dietary Guidelines for Americans 2015–2020, within the context of a poor diet quality (low in vegetables, fruits, high in refined grains, and low in whole grains, dairy), certain nutrients, referred to as public health concerns (Calcium, Magnesium, Potassium, Choline, Vitamin A, Vitamin C, Vitamin D, and Vitamin E), are consumed below the EAR or AI level. In our study, adults with a low protein-to-carbohydrate ratio had poor diet quality with a lower intake of the nutrients mentioned above.

Our study presents new insights into the relationship between %E P:C and diet quality assessed by HEI-2015 and TNI. According to our findings, %E P:C reflects the total nutrient exposure, especially of under-consumed nutrients reported in the latest DGA. It also provides a holistic understanding of the overall diet quality of participants according to their protein and carbohydrate intake. Vitamins, minerals, and foods found to be lower in those with low %E P:C are well-known for their physiological functions in human metabolism.

In the context of poor diet quality, low %E P:C and low %E P is associated with an increased risk of death from all-cause. When analyzing protein intake with all-cause mortality in the isocaloric model, although low intake was associated with a significantly increased risk of death, the risk was lower than that of %E P:C. There is little evidence of the nutritional context within the association between protein-to-carbohydrate intake ratio and all-cause mortality. Findings pointing toward the harmful, beneficial, or null associations between protein intake and health outcomes are inconsistent. A recent study assessed protein intake irrespective of source with all-cause and cause-specific mortality, wherein low protein intake was associated with an increased risk of all-cause and cause-specific death (32). Another study on protein intake and mortality among 7,447 men found a non-linear relationship between protein intake and mortality, where the low intake was associated with higher mortality risk (33). Lee et al., who investigated the effect of low protein intake on all-cause mortality in the NHANES, also found an increased risk of death with low protein intake (34). Kwon et al. (35), Kelemen et al. (36), and Song et al. (37) found an insignificant link between total protein and all-cause mortality among Koreans, US postmenopausal women, and US health care professionals, respectively. A recent meta-analysis on protein intake also reported evidence from prospective cohort studies suggesting that total protein intake is positively associated with all-cause mortality (38). Another meta-analysis of *in vitro* studies established that limiting protein intake prolongs life expectancy (39). Although some of these findings are consistent with ours, the limits of these studies could be the unknown nutritional context in which protein intake leads to their conclusions. In this study, we went a step further to provide more insight into the diet patterns associated with %E P:C impacting the association with all-cause mortality. One unit increase of %E P:C increased the HEI and TNI by 0.22 and 0.16 respectively, implying that the higher the ratio the better the diet quality.

It is beyond the scope of this paper to examine the function of each vitamin, mineral, and food found in lower amounts in those with low %E P:C. However, their physiological functions in human metabolism are well-known. As a result, metabolic homeostasis may be compromised due to habitual insufficiencies of these nutrients with potential consequences of worsening disease outcomes (40–42). Refined grains and sugar are known for their detrimental effect on body composition and metabolic biomarkers (42, 43). Our study also found that in the absence of carbohydrates, low protein intake was still associated with an increased risk of death. Pezeshki et al., assert that low protein diets produce divergent effects on energy balance, and that inadequate nutrition is linked with low protein diets (42). In the same study, inadequate protein intake decreased plasma concentrations of multiple essential amino acids, and metabolic hormones (42). Essential amino acids play a crucial role in sustaining skeletal-muscle protein synthesis, mass, and function (including physical strength) while improving insulin sensitivity, ameliorating aging-associated diseases, and reducing white fat accretion (44).

The poor diet quality and low intake of essential micronutrients, associated with low %E P:C in our study, is linked to inflammatory mechanisms underlying many complex diseases which increase disease risk and death from all-cause (45). Such a diet is often linked with various metabolic disorders, increased oxidative stress (46–48), altering blood pressure, lipid profile (49, 50), and reduced insulin sensitivity (9). It also increases the risk of T2DM (1), hypertension (50), metabolic syndrome, coronary heart disease, and cardiovascular diseases (51), decreased mobility, increased risk of falls and fractures (52), and some cancers (45). Nieuwenhuizen et al. and Pilgrim et al. report that a higher likelihood of lower micronutrient intakes (on the day of intake) and nutrient deficiencies might increase disease risk and reduce life expectancy (1, 53, 54). Hence, in the context of poor diet quality, low %E P:C is associated with an increased risk of death from all-cause, as our findings suggest.

Our study has important strengths. First, it is the first to assess the dietary context (HEI and the TNI) associating %E P:C and all-cause mortality. Previous studies assessed protein intake with health outcomes ignoring that protein intake is part of a complete diet. Second, we used a good nationally representative survey with high-quality dietary data from a well-designed population-based study (NHANES), strengthening the understanding and the impact of food consumption on health. However, our study has a number of limitations. First, the diet was assessed based on the 24-h dietary recall, prone to omissions of food items and therefore may not represent habitual dietary behavior. Nevertheless, it is the most valid and commonly used instrument to capture diet information in observational studies. Second, the causality could not be established because of the observational study design. Third, the study only included American adults, limiting our findings' generalizability to other populations.

## Conclusion

Our findings indicate that %E P:C is positively associated with diet quality. A low protein-to-carbohydrate ratio that gives emphasis to unhealthy diets increases the risk of death. These findings also suggest that a diet's quality can be determined by its protein-to-carbohydrate ratio. Hence, it would be useful to consider the complete diet associated with high or low protein intake when making dietary recommendations for populations. Longitudinal studies are needed to further assess the nutritional context in which %E P:C long-term changes associates with all-cause mortality.

## Data availability statement

All data and materials are publicly available at the National Health and Nutrition Examination Survey, accessible at: https://wwwn.cdc.gov/nchs/nhanes/Default.aspx.

## Ethics statement

The NHANES III is reviewed and approved by the National Center for Health Statistics (NCHS) Institutional Review Board (IRB). The NCHS IRB (after 2004)/and the NCHS Research Ethics Review Board (ERB) (the year 2007–2010) approved the NHANES 2005–2006 (Protocol #2005–06 for the NHANES 2005–2010 and protocol #2011–17 for the NHANES 2011–2017). Prior to the data collection, informed consent was obtained from participants.

## Author contributions

TW: conceptualization, methodology, data analysis, and writing—original draft. RN, YW, and WW: reviewing and editing. SZ: supervised the research. All authors critically revised, read, and approved the final manuscript.
