# Supplementary material for: Protein-to-carbohydrate ratio is informative of diet quality and associates with all-cause mortality: Findings from the National Health and Nutrition Examination Survey (2007–2014)
Source: Front Public Health. 2022 Dec 22;10:1043035. doi: 10.3389/fpubh.2022.1043035 (PMC9814161; doi:10.3389/fpubh.2022.1043035)
Supplement: Supplementary file 1 [file Table_1.docx]

| **Supplementary Tables 1: Hazard ratio (95% confidence interval) of all-cause mortality for %E P: C *z-score* quintiles after excluding participants younger than 30 years old who died within three years of follow-up.** | | | | | | |
| --- | --- | --- | --- | --- | --- | --- |
|  | Model 1 | | Model 2 | | Model 3 |  |
|  | HR | 95%CI | HR | 95%CI | HR | 95%CI |
| Q1 | 1.98 | 1.44-2.26 | 1.78 | 1.33- 2.40 | 2.42 | 0.54-10.81 |
| Q2 | 1.92 | 1.39-3.16 | 1.73 | 1.23-2.44 | 8.48 | 1.88- 38.24 |
| Q3 | 1.84 | 1.35-2.52 | 1.78 | 1.29- 2.44 | 8.14 | 2.50-26.50 |
| Q4 | 1.70 | 1.18-2.46 | 1.58 | 1.07-2.32 | 3.61 | 0.90-14.51 |
| Q5 | 1 | Ref | 1 | Ref | 1 | Ref |
| Model 1: Unadjusted  Model 2: Age, Sex, Ethnicity  Model 3: Model2+Education level Family income, BMI, METs, alcohol consumption, Smoking status, Diabetes medications, Hypertension medications, SBP, Sodium intake, Fat percentage energy, total energy. | | | | | | |

| **Supplementary Tables 2: Hazard ratio (95% confidence interval) of all-cause mortality for energy from Protein z-score quintiles+ Isocaloric regression model after excluding participants younger than 30 years old who died within three years of follow-up.** | | | | | | |
| --- | --- | --- | --- | --- | --- | --- |
|  | Model 1 | | Model 2 | | Isocaloric model | |
|  | HR | 95%CI | HR | 95%CI | HR | 95%CI |
| Q1 | 1.41 | 0.90- 2.20 | 1.38 | 0.91-2.10 | 0.80 | 0.27-2.38 |
| Q2 | 1.54 | 1.06-2.23 | 1.66 | 1.12-2.48 | 2.00 | 0.84-4.74 |
| Q3 | 1.60 | 1.08-2.37 | 1.51 | 1.02-2.25 | 2.50 | 1.17-5.37 |
| Q4 | 0.91 | 0.59-1.41 | 0.95 | 0.61-1.49 | 2.20 | 0.95-5.10 |
| Q5 | 1 | Ref | 1 | Ref | 1 | Ref |
| Model 1: Unadjusted  Model 2: Age, Sex, Ethnicity  Isocaloric model: Model2+Education level, Family income, BMI, METs, Alcohol consumption, Smoking status, Diabetes medications, Hypertension medication, SBP, Sodium intake, Fat percentage energy, Total energy. | | | | | | |
